# Heme-albumin: an honorary enzyme

**DOI:** 10.1038/cddis.2015.287

**Published:** 2015-10-08

**Authors:** P Ascenzi, A di Masi, G Fanali, M Fasano

**Affiliations:** 1 Interdepartmental Laboratory for Electron Microscopy, Roma Tre University, Roma I-00146, Italy; 2 Department of Sciences, Roma Tre University, Roma I-00146, Italy; 3 Department of Theoretical and Applied Sciences, Biomedical Research Division, University of Insubria, Busto Arsizio I-21052, Italy; 4 Center of Neuroscience, University of Insubria, Busto Arsizio I-21052, Italy

Human serum albumin displays time-dependent heme-based catalytic properties,^1^ representing a case for ‘chronosteric effects'.^2^ In fact, HSA has a pivotal role in heme transfer from high- and low-density lipoproteins to hemopexin. After endocytosis of the hemopexin-heme complex into the hepatic parenchymal cells through the CD91 receptor, hemopexin releases the heme, which undergoes degradation. Then, hemopexin is released intact into the bloodstream.^3, 4^

The three-domain organization of HSA is at the root of its extraordinary ligand-binding capacity and allosteric control. The most relevant clefts hosting ligands are the so-called fatty acid (FA) binding sites (named FA1 to FA9). Bacterial protein-recognition cleft(s), thyroxine-binding pockets and metal ion-recognition sites also participate to HSA actions.^4^ Noteworthy, the HSA structure and reactivity is affected not only reversibly by pH and ligands (e.g., heme, FAs and drugs), but also irreversibly by chemical modifications, which in turn confer antigenicity properties.^4^

Ferrous human serum heme-albumin (HSA-heme-Fe(II)) binds reversibly to NO and CO. Although the heme-Fe atom of HSA-heme-Fe(II) is rapidly oxidized by O_2_, HSA-heme-Fe(II) mutants bearing residues pivotal for O_2_ recognition have been proposed not only as red blood cell substitutes, but also as O_2_-therapeutic agents.^1, 4, 5^ Moreover, HSA-heme-Fe(II) catalyzes the nitrite conversion to nitrogen monoxide under acidosis and anaerobic conditions,^6^ HSA-heme-Fe(II)-NO reacts with O_2_ and peroxynitryte leading to the formation of NO_3_^−^,^7, 8^ and ferric HSA-heme-Fe (HSA-heme-Fe(III)) catalyzes the conversion of peroxynitryte to NO_3_^−^,^9^ and displays weak catalase and peroxidase activities.^10^

The heme-based catalytic properties of HSA are allosterically modulated by drugs (Figure 1).^1^ Domains I and II have a major role in the allosteric modulation of ligand-binding and reactivity properties of HSA, the FA1, FA2, FA6 and FA7 sites being functionally linked. Allosteric modulators (e.g., drugs) of the heme-based catalytic properties of HSA-heme affect the coordination state of the heme-Fe atom. In ligand-free active HSA-heme, the heme-Fe atom displays a four- or five-coordinated heme-Fe atom, whereas inactive HSA-heme shows a six-coordinated heme-Fe atom. Upon drug binding to HSA-heme (most probably to the FA2 site), the re-orientation of the Glu131-Arg145 *α*-helix and the axial coordination of the heme-Fe atom by His146 and Tyr161 occur; as a consequence, the unreactive six-coordinated HSA-heme species becomes predominant.^1, 11, 12^

As a whole, the allosteric modulation of heme-based reactivity properties of HSA-heme by drugs represents a pivotal issue in the pharmacological therapy management, heme-binding switching HSA from a plasmatic carrier to a transient metal-enzyme.

## Figures and Tables

**Figure 1:**
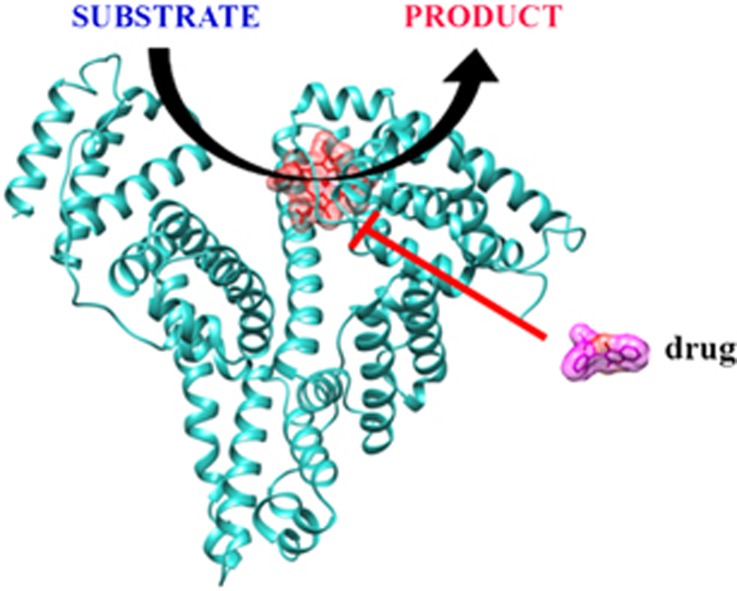
Human serum albumin displays time-dependent heme-based catalytic properties, which are allosterically modulated by drugs
